# From cultural dispositions to biological dimensions: a narrative review on the synergy between oral health and vitamin D through the lens of Indian habitus

**DOI:** 10.3389/froh.2025.1569940

**Published:** 2025-04-25

**Authors:** Sumit Maitra, Hari Charan Behera, Arkopala Bose, Diptendu Chatterjee, Arup Ratan Bandyopadhyay

**Affiliations:** ^1^ Department of Anthropology, University of Calcutta, Kolkata, West Bengal, India; ^2^ Sociological Research Unit, Indian Statistical Institute, Giridih, Jharkhand, India

**Keywords:** periodontal health, vitamin D deficiency, traditional healthcare practices, social space, ethnomedicine, health policy

## Abstract

Oral health is intricately related to systemic health, with new worldwide research demonstrating vitamin D's critical role in sustaining dental and periodontal health. Vitamin D regulates calcium and phosphate metabolism, which is required for the formation and maintenance of healthy teeth and bones. According to research, vitamin D deficiency may contribute to the etiology of periodontal disease by decreasing the host immune response, making it more susceptible to infections like gingivitis and periodontitis. Oral health in India is a tapestry of traditional practices, socioeconomic factors, lifestyle factors, and access to modern healthcare, all of which are intricately linked with the concept of habitus, which refers to deeply embedded habits, dispositions, and practices shaped by an individual's social space. Deep-rooted social and cultural influences have a substantial impact on oral hygiene practices, food patterns, and health-seeking behaviours. Oral diseases are considered as a worldwide health issue. Though standard Western medicine has had effectiveness in preventing and treating periodontal diseases and other oral disorders, the hunt for alternative solutions continues, and natural phytochemicals extracted from plants used in traditional medicine are regarded as viable alternatives to synthetic chemicals. India's traditional medical knowledge and practice, take a comprehensive approach to oral health, emphasizing the balance of physiological components and the use of natural treatments to maintain oral hygiene and treat oral disorders. However, the structural integrity of teeth and optimal oral health can be accomplished by combining Indian traditional medical practices with vitamin D supplementation, which has synergistic attributes for gum health, anti-inflammatory effects, and dental caries prevention. Nevertheless, the unique association of oral health, vitamin D deficiency and the habitus from Indian perspective is extremely underrepresented in academia. To the best of our knowledge, in the aforementioned context, the present narrative review is probably the maiden attempt to discern the crosstalk of oral health and Vitamin D from the perspectives of Indian habitus.

## 1 Introduction

The term “health” encapsulates an individual's comprehensive and state of physical, mental, and social well-being and not merely the absence of disease or infirmity (1). Oral health plays an indispensable role in overall systemic health. Optimal oral hygiene enables individuals to maintain their social appearance and confidence through functional and aesthetically pleasing dentition. Conversely, compromised dental health adversely affects speech, mastication, and other oro-facial functions, thereby diminishing social well-being and overall quality of life (2). For example, individuals with mal-aligned dentition may experience social discomfort, leading to avoidance of public interactions and smiling. Poor dental health is linked to systemic conditions such as bacterial pneumonia, cardiovascular diseases, stroke, infective endocarditis, digestive disorders in the elderly, and adverse pregnancy outcomes (3). Evidence suggests a direct association between dental infections, such as periodontitis, and cardiovascular diseases. Oral bacteria can elevate the risk of infective endocarditis and other systemic complications, especially in individuals with predisposing conditions like rheumatic fever or post-organ transplantation (4). Mechanistic pathways include direct microbial effects on endothelial atheroma formation, host-mediated inflammatory responses, and genetic predispositions (5). Furthermore, maternal oral health has profound implications for the oral and systemic health of offspring. Periodontal disease during pregnancy is associated with adverse outcomes such as preterm birth, preeclampsia, low birth weight and nutritional aspects (6). Maternal oral flora transmission to newborns also heightens susceptibility to dental caries in children (7).

Vitamin D, a fat-soluble vitamin, is synthesized endogenously in the skin through ultraviolet B radiation (290–315 nm) and is also obtained from dietary sources (8). Its active metabolite, calcitriol (1,25-dihydroxyvitamin D3), is critical for calcium absorption, bone health, and phosphorus metabolism (9). Beyond its skeletal functions, vitamin D modulates inflammatory processes and exerts immunomodulatory effects by regulating antibacterial defenses, antigen presentation, and adaptive and innate immunity (10, 11). Emerging research highlights the association between vitamin D levels and various oral health outcomes, including periodontal conditions (12), oral cancer (13), tooth mineralization (14), and tooth loss (15). The vitamin D receptor plays a pivotal role in maintaining oral health, particularly in periodontal disease progression (16). Indicators of periodontal health, such as bleeding on probing (BOP), pocket depth (PD), gingival bleeding, clinical attachment loss (CAL), and alveolar bone loss, are closely linked to vitamin D status. Studies demonstrate that higher serum vitamin D levels correlate with reduced periodontal pockets (17), lower risk of tooth loss (10), and improved periodontal health (18).

### 1.1 Synthesis of health and habitus

Extensive research underscores the disproportionate burden of ill health and reduced life expectancy among individuals with lower socioeconomic status compared to their affluent counterparts (19). Contributing factors include limited access to nutritious food (20–22), safety concerns restricting mobility (23–25), inadequate infrastructure and services (26), and psychosocial stressors that exacerbate mental health challenges and substance misuse (27). Conversely, social cohesion, robust networks, and community pride have been shown to promote health and well-being (28). Social epidemiological studies have consistently established strong links between social stratification, class dynamics, and health disparities (29–31). Detrimental social conditions are strongly correlated with oral health challenges (32–34). Determinants such as insufficient family income (35), limited maternal education (36), disrupted childhood education (37), social class (38), and ethnic background (39–41) predispose communities to adverse oral health outcomes.

From a sociological perspective, the concept of habitus, conferred by Bourdieu provides an essential framework for understanding health disparities. Habitus bridges the “objective” and “subjective” dimensions of the social world, elucidating how individuals act and think within their social context without being entirely dictated by social structures (42). In the context of oral health, habitus shapes health-related behaviors such as dietary practices, oral hygiene routines, and health-seeking behaviors, which may vary significantly across different socio-economic groups in accordance with their social space (43). These ingrained practices, beliefs, and lifestyle patterns profoundly influence individual and community health outcomes.

In the Indian context, national oral health surveys remain limited. The Dental Council of India provided its first epidemiological oral health survey report in 2004 (conducted in 2002–2003), followed by another in 2007 (44). In 2016, India's inaugural state-wise evaluation of the global disease burden (GDB) was undertaken (45). However, the critical intersection between oral health and systemic health remains underexplored. Globally, oral diseases continue to constitute a significant public health challenge. The Global Burden of Disease Study, 2017 reported that oral health disorder affects nearly 3.5 billion people worldwide (46). Oral health in India reflects a complex interplay of traditional practices, socioeconomic and lifestyle factors, and access to modern healthcare. This dynamic is deeply intertwined with the concept of habitus, which encompasses ingrained habits, dispositions, and practices shaped by the social milieu. Socio-cultural determinants significantly influence oral hygiene practices, dietary patterns, and health-seeking behaviors. Thus, habitus in India epitomizes the interaction between tradition, economic status, and social environment, shaping both positive and adverse oral health behaviors.

### 1.2 Research gaps in the intersection of oral health, vitamin D, and habitus: a multi-dimensional approach

Despite its critical importance, the nuanced interrelationship between oral health, vitamin D, and the concept of habitus within the Indian socio-cultural context remains underexplored in academic discourse. A nuanced understanding of this intersection can offer valuable insights into how biological, social, and cultural factors converge to shape health outcomes. Previous studies on oral health and vitamin D tends to focus on either biological or epidemiological components, but the larger socio-cultural influences, especially within the Indian context, are often underrepresented and even somehow, overlooked. This research gap calls for a multi-dimensional investigation of how socio-cultural, traditional, and environmental factors influence both oral health and vitamin D status. To the best of our knowledge, this review represents the first such endeavor to explore the coaction between oral health and vitamin D through the sociological lens of Indian habitus. By addressing this junction, we aim to uncover the underlying socio-cultural and environmental determinants that influence oral health behaviors and vitamin D status in India, offering insights for targeted public health interventions and the prospects of future research. Furthermore, this review aspires to bridge existing knowledge gaps by integrating biological, cultural and environmental dimensions, thereby providing a comprehensive and holistic understanding of this multifaceted coaction.

#### 1.2.1 Reconceptualizing oral health: interlinking social well-being, systemic health, and vitamin D

Oral health is often perceived as a subset of physical health, confined to the management of diseases affecting the teeth, gums, and surrounding tissues. However, the scope of oral health extends far beyond mere structural integrity to encompass larger psychosocial elements of well-being. The association between oral health and overall systemic health has long been acknowledged, with research elucidating associations with cardiovascular diseases, diabetes, stroke, and respiratory infections (3). The extent of this linkage has prompted an increasing interest in exploring how social and environmental factors, particularly those shaped by socio-cultural practices, influence oral health outcomes. While the oral cavity serves as a primary entry point for pathogens, it is also a critical indicator of systemic conditions. The persistence of poor oral hygiene, inadequate access to oral care, and untreated dental conditions is often indicative of deeper socio-economic and cultural determinants. Notably, the burden of oral diseases disproportionately impacts disadvantaged populations, linking social inequality with an increased vulnerability to poor oral health and systemic diseases (47). Oral health is often studied from a biomedical perspective, emphasizing clinical diagnostics and treatments for conditions like cavities, gum disease, and tooth loss. While this approach is crucial in understanding the physiological factors contributing to oral diseases, it largely overlooks the socio-cultural determinants that influence oral health behaviors, particularly in the context of the Global South (48). Dietary habits, oral hygiene practices, and health-seeking behaviors are deeply shaped by social, cultural, and economic factors, making it essential to incorporate these elements into oral health research (49). In India, the role of oral hygiene practices such as chewing sticks, herbal powders, and oil pulling remains a topic of interest but is not yet explored in depth from a socio-cultural or habitual standpoint (50, 51). While the traditional practices of rural and urban communities are often seen as archaic, they represent a culturally ingrained habitus that shapes individuals' responses to oral health issues. Oral health disparities are evident across different socio-economic strata, with marginalized communities facing barriers to accessing modern dental care.

Nevertheless, India's rich socio-cultural heterogeneity significantly shapes oral health behaviors, with region-specific practices, beliefs, and indigenous knowledge systems forming the foundation of daily hygiene routines. In a study conducted among ethnic tribes in Assam's Nalbari and Barpeta districts, Deka and Nath documented the use of 39 plant species for oral health care—such as *Azadirachta indica* (neem) and *Salvadora persica* (miswak) for tooth cleaning, and *Mimusops elengi* (bakul) leaves for mouth rinsing—reflecting a deeply embedded traditional health system (52). Similarly, research by Asif et al. among tribal children in Bhadrachallam in the Eastern Ghats, reported that the use of twigs and other indigenous materials for oral hygiene yielded outcomes comparable to those using toothbrushes and toothpaste, highlighting the efficacy of culturally rooted practices (53). In Maharashtra's Nandurbar district, tribes such as the Bhills, Gavits, and Koknas utilize medicinal plants like *Azadirachta indica* (neem) twigs for dental hygiene and *Acacia catechu* (khair) bark for treating tooth decay (54). Similarly, the Yanadi tribe in Andhra Pradesh employs herbs including *Ocimum sanctum* (holy basil) and *Curcuma longa* (turmeric) for managing oral ailments (55). In Odisha's Nuapada district, ethnic communities also use *Azadirachta indica* (neem) for its antimicrobial properties in periodontal care (56). These empirical findings emphasize that oral health in India cannot be understood in isolation from traditional and cultural context. Region-specific approaches that integrate traditional practices with modern dentistry can improve oral health outcomes while preserving indigenous knowledge. These studies revealed that communities often rely on folk remedies and traditional knowledge passed down through generations, which may or may not align with evidence-based clinical practices. Furthermore, the relationship between oral health and systemic health has gained increasing recognition in recent years, especially the connection between periodontal disease and chronic conditions such as diabetes and heart disease (57–61). However, oral health's role in overall well-being is still often perceived through a narrow biomedical lens, which fails to address the broader social context in which oral health behaviors are embedded.

Vitamin D plays a critical role in bone health, including the health of teeth, and its deficiency is linked to various conditions such as osteomalacia, rickets, and periodontal disease (62–64). Despite growing recognition of vitamin D's impact on health, research in India often fails to connect vitamin D status with social and cultural practices, particularly diet and sun exposure behaviors (65). The main sources of vitamin D-sunlight and dietary intake-are heavily influenced by cultural practices, particularly in sun exposure and dietary habits (66, 67). In certain Indian communities, dietary patterns are shaped by religious beliefs, socio-economic status, and geographical variations. For example, vegetarian diets may lack sufficient vitamin D, which is predominantly present in animal-based products like fish, eggs, and fortified dairy products (68, 69). Additionally, cultural practices, such as veiling or limited outdoor activities in conservative households, can drastically limit sun exposure, an essential factor for vitamin D synthesis (70). Moreover, the regional disparity in vitamin D levels across India further complicates the situation (71). For instance, individuals in northern parts of India, where sunlight is limited in the winter months, are often found to have lower levels of vitamin D compared to those living in southern parts, where sunlight is more consistent year-round (72, 73). These regional differences are shaped by habitual behaviors that are both culturally and economically influenced, presenting a critical research gap in understanding how these cultural norms impact vitamin D status. The urban-rural divide, coupled with varying dietary habits, lifestyle practices, and access to sunlight, plays a critical role in determining an individual's vitamin D levels. Research has shown that individuals in rural areas often exhibit lower serum vitamin D levels due to limited access to fortified foods, inadequate exposure to sunlight, and socio-economic challenges that hinder their awareness about vitamin D deficiency and ability to access further medical intervention. Moreover, factors such as clothing patterns (influenced by regional, cultural, and religious practices) can further restrict sun exposure, exacerbating vitamin D deficiency, especially among women (74). The health implications of vitamin D deficiency are far-reaching. Inadequate levels of this vital nutrient have been associated with a higher incidence of chronic diseases, including osteoporosis, cardiovascular diseases, and autoimmune disorders. For oral health, vitamin D plays a climacteric role in calcium metabolism and the prevention of periodontal diseases. Studies have established a clear correlation between low vitamin D levels and the severity of periodontal disease, with vitamin D deficiency contributing to inflammation and alveolar bone loss (75, 76). Given that India has one of the highest rates of vitamin D deficiency globally, identifying the socio-cultural determinants of vitamin D status is vital for formulating effective public health interventions (77). The crucial role of vitamin D in oral health has also been a subject of intense scrutiny, as it is central to calcium metabolism, bone health, and immune function. Recent studies have established a bidirectional relationship between vitamin D deficiency and oral diseases, particularly periodontitis, caries, and tooth loss. The modulation of immune responses through vitamin D's effects on the innate and adaptive immune systems has made it a key factor in the regulation of periodontal health.

The concept of habitus, as defined by Pierre Bourdieu, refers to the profoundly embedded and enormously ingrained habits, skills, and dispositions that individuals develop through their life experiences, particularly within specific social contexts. Habitus shapes individuals' behaviors, attitudes, and perceptions, often beyond their conscious awareness, and thus plays a pivotal role in determining health behaviors, including those related to oral hygiene and health-seeking practices. In the context of oral health, habitus influences not only individual health practices-such as brushing, dietary choices, and the seeking of dental care, but also societal norms that govern health practices. This framework is pertinent to fathom the inequities in healthcare system where structured barrier, e.g., economic disparities, cultural stigmas, and discrepancy in awareness intersect with individual practices to perpetuate disproportionate effect. Individuals with lower socioeconomic status, for example, may lack access to effective oral healthcare or be less likely to adopt preventive dental practices due to the constraints of their habitus, which is shaped by factors such as income, education, and cultural values (78, 79). This sociological lens also sheds light on the unequal distribution of oral health outcomes across different social strata. Those with higher socioeconomic status typically have better access to healthcare, superior oral hygiene habits, and greater knowledge about the importance of oral health, which reinforces their health advantages. Conversely, individuals from disadvantaged backgrounds face multiple barriers, including economic limitations, cultural attitudes, and inadequate health infrastructure, all of which contribute to poorer oral health outcomes (80). The health disparities observed across various social classes reveal the profound influence of habitus in shaping individuals' health trajectories. Vitamin D deficiency, which is prevalent in global south when combined with these disparities, aggravate oral health issues, for its critical role in bone metabolism and immune function. This concatenation of oral heath, vitamin D and habitus demands deeper consideration since they directly address the Sustainable Development Goals (SDG3 and SDG10) which advocate for ensuring healthy lives and promoting wellbeing for all and reducing inequalities, particularly accentuating the imperatives to handle discrimination in oral healthcare.

#### 1.2.2 Toward an integrated framework for oral health and vitamin D status in India

As we navigate the complexities of oral health and vitamin D status in India, it becomes evident that a multidimensional approach is necessitated. Bridging the gap between traditional knowledge, contemporary medicine, and social determinants of health is pivotal for developing interventions that are both effective and culturally concordant. Nevertheless, addressing the socio-cultural factors that shape health behaviors-such as education, income, and access to resources can help mitigate the disparities observed in oral health and vitamin D status (81, 82). The concept of habitus, as it relates to oral health and vitamin D, provides a robust framework for understanding the socio-cultural determinants of health. By examining how individuals' social space shape their health behaviors, we can better understand the barriers to optimal oral health and vitamin D levels. The role of habitus in shaping health practices and access to healthcare must be considered in designing interventions aimed at improving health outcomes across diverse social groups. Incorporating these insights into policy and public health initiatives can facilitate the development of effective interventions that promote preventive care and reduce health disparities (80). This exploration of oral health, vitamin D, and habitus underscores the need for a comprehensive and culturally sensitive approach to healthcare in India. By integrating traditional knowledge systems with modern health practices, we can create a more inclusive and holistic model of healthcare that addresses both the biological and socio-cultural aspects of health. Ultimately, the junction of oral health, vitamin D, and habitus provides an opportunity to rethink public health interventions in India, offering some path toward more equitable and effective health outcomes for all.

## 2 Discussions

**Figure 1:**
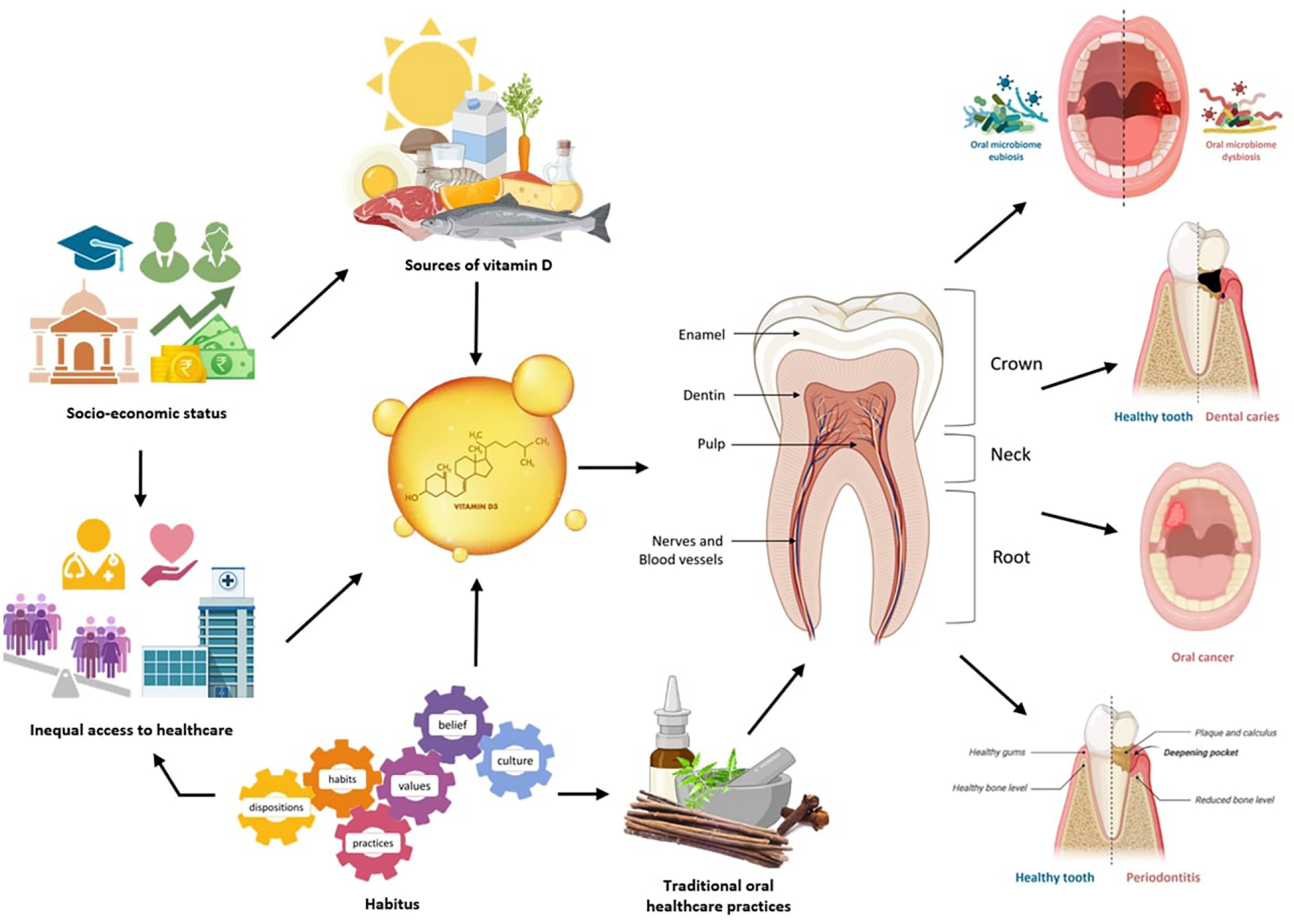
Interconnection between oral health and vitamin D level, orchestrated by habitus in Indian context. Created with BioRender.com.

The present narrative review aims to critically analyze the interconnection between oral health and vitamin D and its relation with habitus in the Indian context. With the analyzed literature, the key socio-cultural, behavioral and biological factors influencing oral health practices, alongside the pivotal role of vitamin D in maintaining oral health can be identified (Figure 1). The review highlights how habitus configured by social, cultural, and economic contexts not only affects individualistic health behaviors, also it enunciates the disparities in access to healthcare, nutritional knowledge, and oral health resources that can be affiliated to socio-economic status and cultural practices of different communities. By understanding these intersections, the potential pathways for improving oral health outcomes in India can be ascertained. This analysis can establish the cornerstone of future research on impact of habitus and nutrition, particularly vitamin D intake on oral health in a holistic manner, contributing to policy and intervention strategies necessary at reducing health inequities in the Indian population.

### 2.1 A closer look at oral health and vitamin D status in India

Healthcare systems bear the critical responsibility of addressing the health needs of their populations in an equitable and non-discriminatory manner. Insights from the 2017 Global Burden of Disease (GBD) study underscore the staggering impact of oral diseases, which afflict an estimated 3.5 billion people worldwide. Among these, untreated dental caries remains the most prevalent condition, eclipsing all other morbidities. Beyond its pervasive presence, oral disease significantly undermines quality of life and imposes substantial financial pressures on public health systems, often exacerbating budgetary constraints. Moreover, the intricate links between oral diseases and chronic non-communicable conditions are well-documented, with tooth loss frequently cited as a marker of increased risk for premature mortality. The psychosocial implications of oral disease, including diminished psychological well-being and social isolation, further magnify its multifaceted burden (47). As India aspires to realize the goal of universal health coverage, strengthening oral healthcare delivery through the recruitment and retention of skilled, motivated dental professionals emerges as a key priority. Developing a robust, data-driven workforce landscape is imperative for crafting effective policies that can reduce the workforce gaps and develop a responsive healthcare system. However, systemic shortages in human resources present formidable challenges, curtailing the scalability of health services and limiting the capacity to translate financial investments into tangible health outcomes. The discourse on health inequalities, particularly their intersections with living conditions, has moved to the forefront of public health scholarship. Paul Farmer's evocative characterization of inequality as the “plague of our era” captures the pervasive and enduring nature of this issue (83). Similarly, Gwatkin's analysis highlights the critical dimensions of ethnicity, gender, and economic status in shaping health outcomes in developing nations (84). Oral diseases persist as a significant public health concern, particularly in rural areas, which house approximately 69% of India's population. The prevalence of both, oral diseases and periodontal diseases are alarmingly high, with different percentages of dental caries in different age groups (47). Comprehensive, equity-focused strategies are essential to mitigating this substantial burden and aligning oral health priorities with broader public health goals.

Vitamin D, a fat-soluble vitamin recognized for its antirachitic properties, belongs to a group of lipid-soluble compounds known as calciferols, characterized by a four-ringed cholesterol backbone. This group includes Vitamin D3 (cholecalciferol) and Vitamin D2 (ergocalciferol). Among these, Vitamin D3 is predominantly referenced in scientific literature. Importantly, Vitamin D can be synthesized endogenously, with approximately 90% of the body's requirement produced in the skin upon exposure to sunlight (85–87). The physiological significance of Vitamin D is extensive, encompassing the maintenance of normal blood calcium and phosphate levels essential for bone mineralization, muscle contraction, nerve conduction, and overall cellular function across all tissues. Furthermore, it plays a vital role in immune regulation, inflammation control, and the processes of cell proliferation and differentiation (62, 87). Endogenous Vitamin D synthesis primarily occurs in the skin when exposed to ultraviolet B (UV-B) radiation within the wavelength range of 290–320 nm. Although dietary sources such as fatty fish, fortified foods, and supplements contribute to Vitamin D levels, they are generally inadequate, particularly as vegetables and grains are poor sources (62). Additionally, various environmental and physiological factors, including geographic latitude, solar zenith angle, atmospheric pollution, ozone layer integrity, and melanin pigmentation, significantly influence the efficiency of cutaneous Vitamin D synthesis (62). The global prevalence of Vitamin D deficiency is alarmingly high, spanning both regions with limited sunlight exposure and those with abundant sunlight availability. Despite this, Vitamin D deficiency remains one of the most underdiagnosed and undertreated nutritional deficiencies worldwide (66, 88). Numerous studies highlight poor Vitamin D status across different age groups, sexes, and geographical regions. However, the lack of universally standardized guidelines for defining Vitamin D deficiency poses challenges in drawing consistent comparisons. Most studies adopt a serum 25-hydroxyvitamin D [25(OH)D] level below 20 ng/ml as the threshold for deficiency, though some employ alternative cutoff values (66, 88). In India, community-based studies conducted over the past decade on apparently healthy individuals have reported Vitamin D deficiency prevalence rates ranging from 50% to 94%. These studies, encompassing various age groups, vividly demonstrate the prodigiousness of the problem (89–105). Hospital-based investigations similarly reveal a prevalence ranging from 37% to 99%, underscoring the widespread and critical nature of Vitamin D deficiency in the country (106–121). Such findings necessitate a comprehensive and context-specific approach to mitigate Vitamin D deficiency through public health initiatives, policy formulation, and targeted interventions addressing both environmental and sociocultural determinants.

### 2.2 Biological underpinnings of oral health and vitamin D

Oral health involving the health of teeth, gums, and their surrounding tissues, reflects one's general health and well-being. The oral cavity is a complex ecosystem hosting a very diversified microbiome, serving as an entrance into the digestive system. Despite its importance, oral diseases such as dental caries, periodontal disease, and oral cancer maintain high disease burdens globally, affecting hundreds of millions of people while imposing substantial economic and quality-of-life burdens. Recent breakthroughs in the understanding of the oral microbiome, host-pathogen interactions, and interplay between oral and systemic health, which, in turn, underlined the urgent need for devising new strategies for the prevention, diagnosis, and treatment of such diseases.

Oral health is indicative of overall bodily health. The effective functioning of the human physiological and biochemical systems necessitates a balanced interaction of various vitamins and minerals. Vitamin D is one such vitamin with pleiotropic effects beyond bone health and is required for maintaining bodily homeostasis (122). Vitamin D is a secosteroid hormone that has long been recognized for its functioning in regulation of calcium and phosphorus homeostasis and bone mineralization. Furthermore, it maintains the balance between different physiological processes involving the skin, musculoskeletal, neuromuscular, and immune systems. Current investigations have been more focused in exploring the antitumor, antibacterial, anti-inflammatory, and other immunomodulatory effects of Vitamin D (123). Teeth, the mineralized organs are consisted of three distinct hard tissues, i.e., Enamel, dentin, and cementum. Teeth are tethered to the alveolar bone that surrounds the tooth socket through an unmineralized periodontal ligament. Vitamin D deficits and genetic abnormalities in vitamin D metabolism effectuate significant alterations in dental-oral-craniofacial structures. In this context, this review attempts to unveil the nexus between vitamin D and oral health.

**Figure 2:**
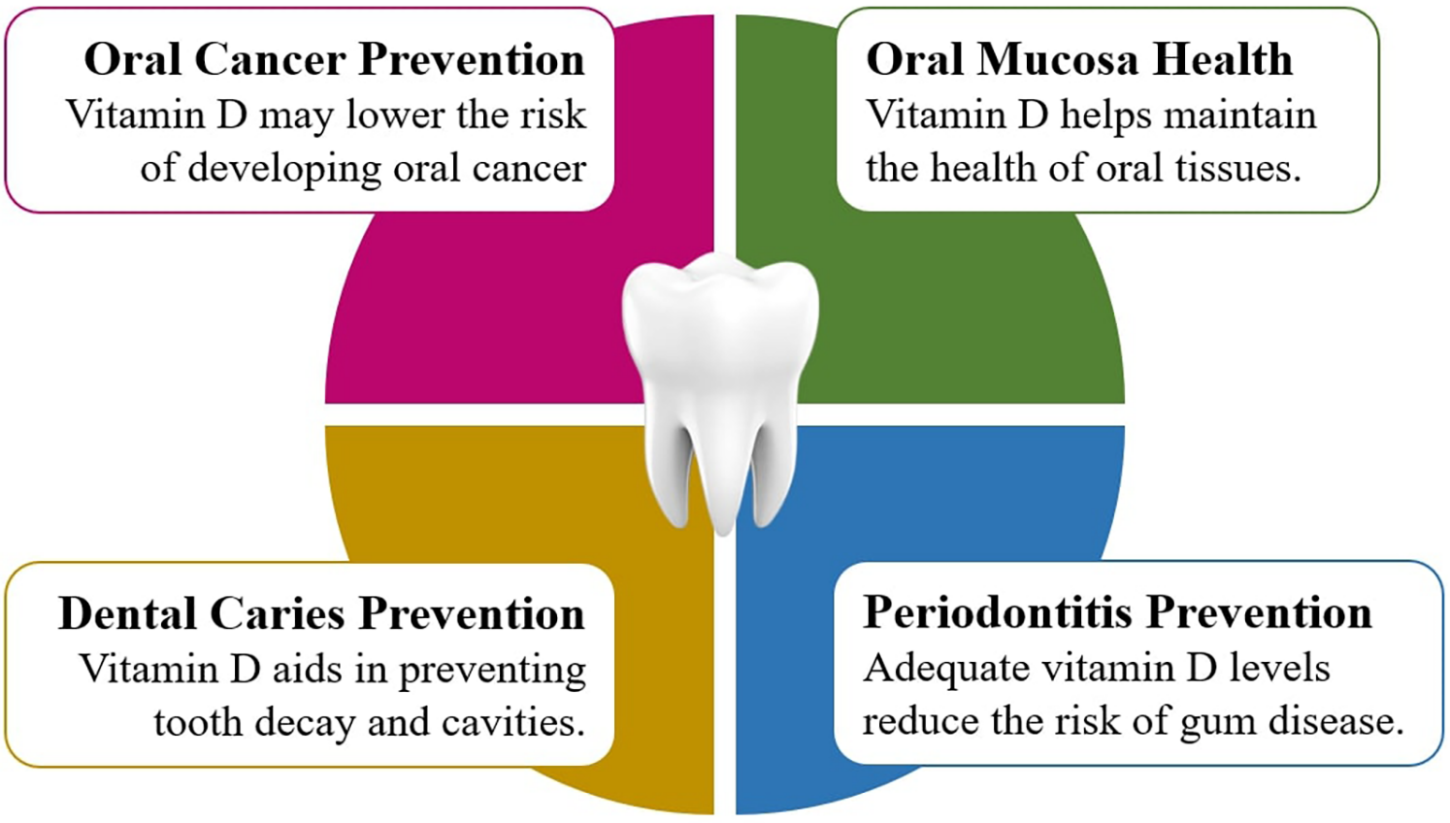
Contribution of vitamin D in mitigating different oral health issues.

Vitamin D is predominantly acquired through solar irradiance, while other supplementary forms include diet and nutritional supplements (124–128). Though intrinsic vitamin D is considered rare in foods, the notable sources include oily fish species such as salmon, mackerel, and herring, together with their oils, like cod liver oil (124). There exist two major bioactive forms of vitamin D, referred to as ergocalciferol, or Vitamin D2, and cholecalciferol, or Vitamin D3. Vitamin D2 is produced by the ultraviolet mediated conversion of ergosterol from yeast, while Vitamin D3 is produced by the irradiation of 7-dehydrocholesterol obtained from lanolin (62, 126, 129) with biological activity equivalent to cholecalciferol. Moreover, Vitamin D3 is endogenously produced in human skin upon sun exposure. Serum 25-hydroxyvitamin D (25[OH]D) levels have been recognized as a widely accepted marker for the measurement of vitamin D status. Essentially, vitamin D is a hormone whose endocrine activity helps to maintain blood calcium and phosphate levels by controlling intestine absorption (62, 124, 130). In addition, it functions as an autocrine and paracrine agent, regulating the innate immune system, cell maturation, and differentiation (131–133). In specifics, the physiologically active form of vitamin D interacts with the VDR, a receptor molecule, to mediate the activities of vitamin D on cell (131, 133, 134). Consequently, the biological functions of vitamin D depend on the modulation of the VDR for its genomic effects and membrane-associated proteins for its nongenomic effects including various signaling pathways (135). This wide activity is explained by the fact that this vitamin controls the expression of a large percentage of genes, and according to estimates, it influences about 5%–10% of the whole genome. The role of nutrition in oral disorders has become very popular and recent studies have pointed out an increasingly important connection between nutritional deficiencies and oral diseases (3, 136). With regards to oral disorders, caries and periodontal diseases are multifactorial diseases of undoubted complexity and continue to be the two most widespread diseases in the world (137, 138). Both caries and periodontal diseases are associated with VDD and its underlying pathophysiologic processes. Mechanisms, which link vitamin D to oral health, are not based only on bone metabolism. Nowadays, research has unveiled that Vitamin D deficiency and its underlying pathophysiologic processes are linked to both periodontal and dental disorders (Figure 2) (139–142). Recent investigations deciphered that vitamin d deficiency disrupts odontogenesis, leading to a hypomineralized dentition that is vulnerable to fractures and carious lesions (142).

#### 2.2.1 Vitamin D protecting mechanism against dental caries

Teeth are mineralized structures made up of three different hard tissues: cementum, dentin, and enamel. They are encased in alveolar bone. Although the mineralization of teeth and skeletons happens simultaneously, problems resulting from disruptions in mineral metabolism will resemble those in bone tissue. A dysregulated levels of vitamin D can result in the “rachitic tooth,” a malformed and hypomineralized structure that is extremely prone to fracture and decay. Vitamin D is essential for the mineralization of bone and teeth. There is a lot of discussion on the methods by which VDD influences tooth mineralization elsewhere (143, 144). The primary biological foundation is based on the observation that severe VDD (<10 ng/ml) results in hypophosphatemia and hypocalcemia, which in turn leads to secondary hyperparathyroidism (caused by hypocalcemia) (145, 146). The resulting hypophosphatemia then deteriorates dramatically. Ultimately, tooth cells with low Ca2+ and phosphate ion concentrations perish their vitamin D signalling pathways, which prevents teeth from properly mineralizing and results in mineralization problems. Circulating vitamin D, in addition to maintaining mineralization equilibrium, can activate a signalling cascade via vitamin D receptors (VDR). Vitamin D-responsive element (VDR) is a ligand-activated transcription factor that regulates the expression of genes. Certain genes that are sensitive, for example, impact bone, immunological response, mineral metabolism, cell migration and life cycle, skeletal muscle, detoxification, and energy metabolism. The production of structural gene products, including as calcium-binding proteins and other extracellular matrix proteins (such as enamels, amelogenins, dentin sialoglycoproteins, and dentin phosphoproteins), can be induced by vitamin D through the upregulation of VDR which enable the formation of dentin and enamel (143, 147).

Maternal 25(OH)D levels can alter deciduous dentition, regardless of inherited foetal abnormalities (148, 149). Vitamin D levels in foetal blood correlate with maternal levels, making it a reliable surrogate marker for the foetus. Therefore, if maternal 25(OH)D levels become imbalanced, this might have a direct impact on the baby's health (150) and, specifically, on tooth formation (148, 149, 151–153). The pattern of mineralization defects depends on the precise week of gestation in which maternal VDD occurred. For instance, around at the 13th week from conception, the human primary maxillary central incisor begins to calcify, and if there is a VDD status, there might be a hypoplasia or mineralization deficit on the incisal third of crown. Recent studies have examined the role of vitamin D supplementation during pregnancy in influencing the dental health of newborns, particularly in the development of deciduous teeth and enamel defects. One randomized controlled trial (RCT) found that pregnant women with serum vitamin D levels below 15 ng/ml exhibited a 14% higher risk of experiencing defects in the deciduous dentition of their offspring (154). Conversely, the administration of high-dose vitamin D supplementation during pregnancy was proven to reduce the odds of enamel defects by approximately 50% (151).

Moreover, untreated caries in permanent and deciduous teeth was the most common ailment, impacting 35% and 9% of the world's population, respectively (155). Caries is also the fourth costliest chronic illness to treat, according to the WHO (156). The pathogenesis of this infectious illness is diverse and intricate. The most extensively researched risk variables were environmental factors such cariogenic bacteria, a diet heavy in carbohydrates, and inadequate dental hygiene (157–159). However, some individuals are more prone to caries than others even when they are exposed to the same environmental risk factors, hence environmental variables by themselves are insufficient to account for the prevalence and incidence of caries. Low vitamin D levels have been linked to a high frequency of caries in both children and adults, although the exact mechanism is yet to be discerned (160–162). Vitamin D is essential for the modulation of immune system activity, influencing both innate and adaptive immune responses. Optimum levels (≥75 nmol/L) are linked to decreased risk of dental caries in children (141, 163, 164). Nonetheless, vitamin D appears to protect caries lesions by regulating the immune system and stimulating antimicrobial efficacy with peptide activity. The effects of UVB are proved to be pivotal in decreasing tooth cavities by producing vitamin D and inducing AMPs, which have antibacterial characteristics (165–168). AMPs are host defensive peptides, mostly cationic and amphiphilic compounds, that provide critical components of innate immunity against a variety of bacteria, fungi, and viruses and help prevent cavities (168, 169).

#### 2.2.2 Role of vitamin D in oral mucosal resistance

The oral mucosal epithelium serves as a critical physical barrier, protecting the soft basal tissue from microbial invasion along with its pathogenic antigens and toxin-producing factors and also from minor physical and structural abrasions (170). The keratinocytes within the basal and spinous layer of the oral epithelium synthesize 1,25-dihydroxyvitamin D (1,25(OH)₂D) and vitamin D receptor (VDR) is expressed within the cells. The crosstalk between 1,25(OH)₂D and VDR regulates the proliferation, differentiation, and apoptosis of the keratinocytes. This signalling pathway also influences immune responses within the epithelial layer, influencing their sensitivity to various stimuli (171).

Vitamin D and VDR ally together to enhance the antimicrobial immune response of the innate immune cells which activate adaptive immunity when exposed to virulence through antimicrobial peptides (AMPs). VDR is expressed in both the cells of innate and adaptive immune systems. Additionally, a few cells also express CYP27B1 and synthesize the physiologically active 1,25(OH)₂D. Upon the activation of Toll-like receptors (TLRs) to recognize the pathogen-associated molecular patterns (PAMPs) and subsequent modulation of immune response by natural immune cells augments the expression of CYP27B1 and VDR, and results in synthesis of 1,25(OH)₂D. The AMP genes β-defensin 2/defensin-β4 (HBD2/DEFB4) and cathelicidin antimicrobial peptide (CAMP) are encoded by 1,25(OH)_2_D/VDR signalling in the immune cells, thus contributing to the innate immune defence (172, 173). The 1,25(OH)_2_D/VDR signalling pathway contributes to oral tolerance by modulating the function of regulatory T cells (Tregs), in maintaining immune homeostasis. 1,25(OH)_2_D downregulates the maturation of antigen-presenting cells (APCs), particularly dendritic cells (DCs), by binding to the VDR. This leads to their decreased capacity to present antigens as peptides to T cells on human leucocyte antigen (HLA) molecules and subsequent diminished T cell activation and proliferation, potentially leading to T cell anergy and altered proinflammatory cytokine production (12, 172, 174, 175).

#### 2.2.3 Impact of vitamin D on progression of oral carcinogenesis

Vitamin D exerts an oncostatic effect on pathogenesis of certain oral cancer, though the researches are still at a nascent phase. Research suggests vitamin D deficiency (VDD) increases susceptibility to cancer progression, and vitamin D and its bioactive analogues have potential therapeutic and cancer-inhibiting features. Cancerous tissues demonstrate elevated expression of the vitamin D receptor (VDR), and both *in vivo* animal models and *in vitro* cell culture studies have shown that the active form of vitamin D, 1,25(OH)₂D, inhibits critical processes such as cell proliferation, angiogenesis, and tumour invasion, while also promoting cellular differentiation and apoptosis. This antitumorigenic effect is mediated through the activation of cyclin-dependent kinase inhibitors specifically p21 & p27, which disrupt the action of mitogenic growth factors such as insulin-like growth factor 1 (IGF-1) and epidermal growth factor (EGF), while simultaneously enhancing the tumour-suppressive effects of transforming growth factor-beta (TGF-β). These actions collectively contribute to the inhibition of cancer cell proliferation and tumour progression. Additionally, the 1,25(OH)₂D/VDR signalling axis plays a pivotal role in modulating the inflammatory pathways associated with cancer by downregulating cyclooxygenase-2 (COX-2), prostaglandin synthesis, and NF-kB signalling. This results in the inactivation of antiapoptotic proteins, such as Bcl-2, and the activation of proapoptotic factors, such as Bax and RAK, thereby promoting apoptosis of malignant cells (175, 176). Patients of oral cancer invariably exhibit a deficiency in their serum vitamin D levels. A case-control study demonstrated that vitamin D has escalating impact on squamous Oral, pharyngeal, and oesophageal cell carcinomas among and those with severe smoking and alcoholism (177). Different research indicated, vitamin D supplementation significantly mitigate chemotherapeutic toxicities in advanced stage oral malignancies, which leads to a reduction in morbidity and an enhanced overall quality of life. In addition, VDR is over-expressed in both premalignant lesions and oral neoplasms (178).

#### 2.2.4 Influence of vitamin D in the prevention and management of periodontal diseases

Periodontitis ranks among the most widespread global health conditions, with its advanced stage classified as the sixth most prevalent, causing significant social, economic, and systemic burdens that affects individual's quality of life (179, 180). Periodontitis, a multifactorial and polymicrobial complex inflammatory disease is found to be exacerbated in presence of various other health conditions, including diabetes, ischemic stroke, cardiovascular disease, rheumatoid arthritis, inflammatory bowel disease, stress, complications in solid organ transplant recipients, and preterm birth (181). In alignments with these systemic connections, extensive research has explored the impact of nutrition in periodontal health, specifically the influence of vitamin D deficiency. Recent European consensus guidelines emphasize that insufficient vitamin D levels can have a detrimental effect on periodontal health and oral functionality (3). The periodontal infections trigger both inflammatory and immunological responses of host immune system with elevated levels of IL-35, IL-17A, and transforming growth factor, indicating an inflammatory environment to periodontal infections (182) and a decreased level of salivary concentration of vitamin D have been associated with high levels of inflammatory biomarkers in individuals with periodontitis. Inflammatory stimuli trigger the dental pulp fibroblasts and periodontal cells to enhance to local biosynthesis of 1,25(OH)₂D. This active metabolite of vitamin D facilitates immunoprotective response by binding to VDR and activating non-specific as well as specific immune system necessary to mitigate microbial load within oral microenvironment (75). Numerous research revealed low serum vitamin D concentration is closely linked with aggravated periodontal inflammation and alveolar bone resorption. The immunomodulatory effect of Vitamin D signalling pathway in periodontal soft tissues primarily mediated by the upregulation of cathelicidin, a key cationic antimicrobial peptide integral to innate immune system. drive a key human cationic antimicrobial peptide integral to innate immune responses (183, 184). Hitherto, cross-sectional investigations of gingival samples reveal reduced level of VDR, along with less fibroblast cells but more inflammatory cellular infiltrates in comparison to healthy persons (185).

### 2.3 From structure to symptoms: interrogating Bourdieu's concept of habitus in the context of vitamin D and oral health in India

Pierre Bourdieu's concept of habitus represents a theoretical framework that encapsulates the interplay between societal structures and individual agency. Defined as a set of durable, transposable dispositions, habitus is shaped by historical and social contexts and, in turn, shapes human behaviors and practices (186). This notion underscores the embodiment of social structures within individuals, manifesting in habitual practices, preferences, and lifestyle choices. In the Indian context, habitus acquires a nuanced dimension, interwoven with caste, class, ethnicity, and regional identities, influencing health behaviors and access to resources.

The Indian habitus reflects deeply embedded norms and values shaped by caste, religion, and community affiliations. These elements dictate dietary practices, physical activity levels, and exposure to sunlight, factors intrinsically linked to vitamin D levels. Vitamin D deficiency in India is paradoxical, given the country's abundant sunlight. However, cultural, dietary, and lifestyle practices contribute significantly to this issue. For instance, traditional attire, especially among women in conservative communities, limits sun exposure, contributing to vitamin D deficiency (187, 188). Traditional Indian diets common in certain populations, often vegetarian or predominantly plant-based, lack sufficient natural sources of vitamin D such as fatty fish, eggs, and fortified foods (73). While ghee and fermented dairy products like buttermilk are consumed in some regions, their contribution to vitamin D levels is minimal.

**Figure 3:**
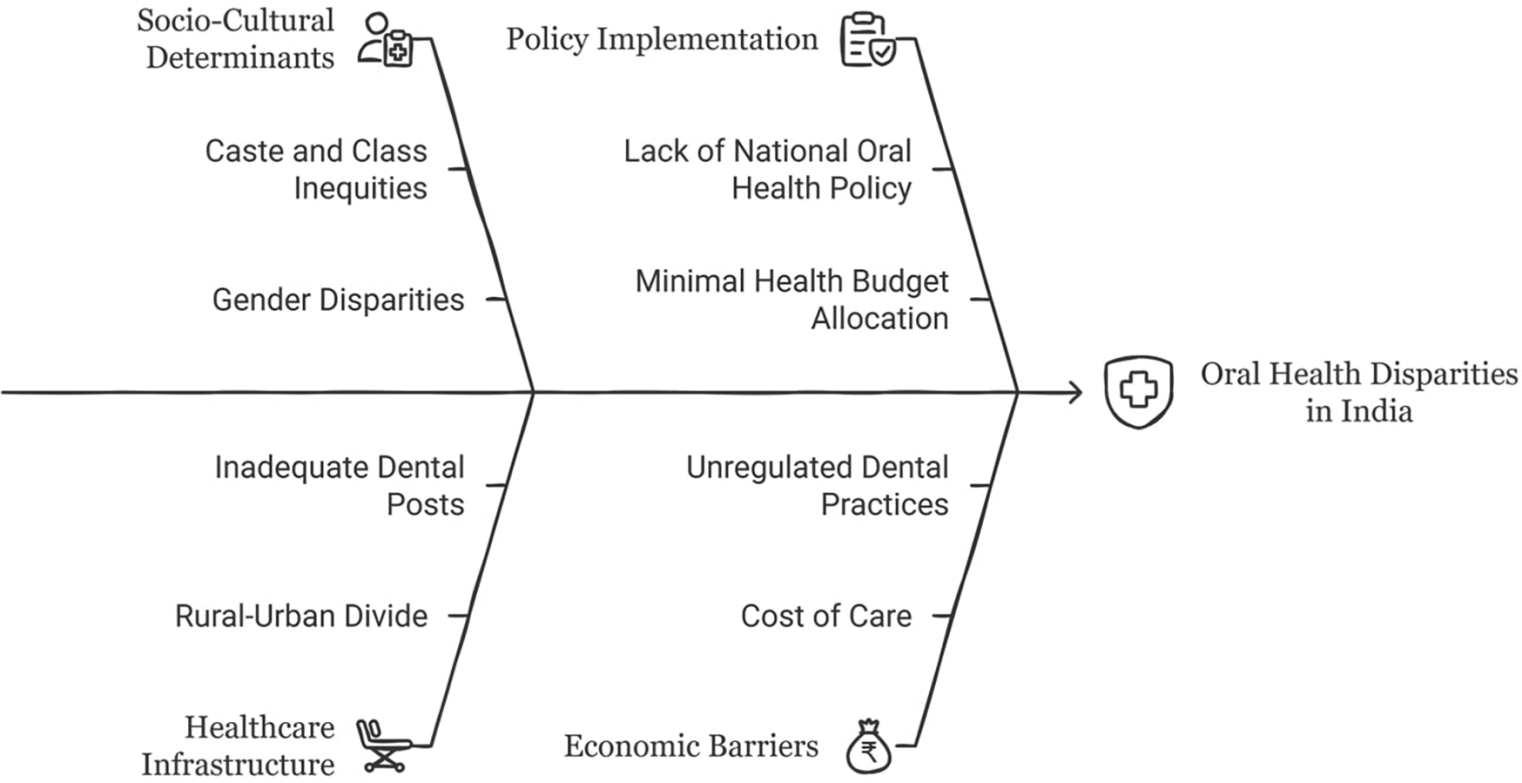
Addressing oral health disparities India.

Bourdieu's concept of *habitus* offers an insightful lens to analyze the structural and embodied dimensions of oral health disparities in India (Figure 3). Far from being a mere constellation of individual preferences or lifestyle choices, *habitus* is an embodied history-a durable, generative matrix of dispositions produced by one's social conditions (78). These dispositions unconsciously guide practices, perceptions, and valuations, including those related to health and hygiene. Among marginalized communities in India, oral health behaviors are not merely consequences of ignorance or negligence, but are deeply structured by material deprivation, cultural capital asymmetries, and institutional exclusion from formal healthcare systems (189).

#### 2.3.1 Economic and cultural capital: the structural determinants of oral health disparities

Bourdieu's notion of capital (economic, cultural, and social) provides an effective framework to elucidate the stratified access to oral healthcare. Economic capital determines the affordability of preventive and curative dental services, often rendering such care a luxury for low-income groups. In communities where formal dental care is economically prohibitive, reliance on traditional practices such as *datun* (neem twigs), charcoal, or salt emerges not from cultural preference but from structural necessity (190). These practices become *doxic*, taken-for-granted norms reproduced within social fields and misrecognized as tradition, thereby obscuring their roots in deprivation (191).

Cultural capital, particularly in the form of health literacy, further reinforces these divides. Individuals embedded in middle-class or elite contexts, armed with biomedical knowledge and institutional trust, are more likely to incorporate routine oral hygiene and preventive care into their everyday lives (192). Conversely, marginalized groups, lacking this symbolic capital, deprioritize oral health or perceive it as irrelevant until crisis thresholds are reached. This divergence in health behavior becomes a site of *symbolic violence*—a process whereby social domination is internalized and misperceived as natural, fostering self-blame and obscuring the structural roots of inequality (193, 194).

#### 2.3.2 Symbolic violence and the normalization of dental neglect

The normalization of oral pain and dental neglect among socially excluded groups further exemplifies how symbolic violence operates through habitus. In the absence of accessible, empathetic care, chronic pain and tooth loss are often seen as inevitable rather than preventable. This embodied resignation is less a reflection of apathy than a conditioned response to sustained institutional marginalization. Dental clinics, often perceived as alienating spaces of biomedical authority, reinforce this dynamic. Lower-income patients frequently report feelings of inadequacy, shame, or intimidation in these settings, exacerbated by hierarchical interactions with medical professionals (195). Such experiences foster alienation and medical avoidance, deepening cycles of oral health neglect.

#### 2.3.3 Disrupting the cycle: beyond behavior to structural interventions

Addressing these disparities requires a paradigmatic shift from behavior-centric to structure-aware interventions. Public health strategies must foreground equity by subsidizing dental care, integrating oral health into primary care systems, and deploying mobile dental units in underserved areas. Additionally, health promotion efforts must cultivate *critical health literacy*—empowering individuals to question doxic assumptions and navigate complex healthcare systems (196). Culturally competent education programs should respect traditional oral practices while demystifying biomedical norms, ensuring that health knowledge is co-produced rather than imposed.

In essence, applying Bourdieu's *habitus* to oral health in India reveals that disparities are less about individual choices and more about historically sedimented dispositions shaped by economic scarcity, cultural marginalization, and symbolic domination. Remediating these inequalities demands not only institutional reform but also the sociological reconfiguration of how oral health is conceptualized, accessed, and valued.

#### 2.3.4 From disposition to disease: the socio-structural anatomy of oral habitués

The triadic interplay of *habitus*, *habits*, and *habitués* offers a compelling framework for understanding the embodiment of health-related behaviors. Rooted in Bourdieu's sociological theory, *habitus* signifies the internalized dispositions shaped by historical and cultural conditions, which unconsciously guide individual practices. These dispositions emerge as *habits* (repetitive and routinized actions), that, over time, give rise to *habitués*: persistent and often deleterious engagements such as tobacco or alcohol use. This continuum underscores how socio-cultural structures inscribe themselves onto the body through practice, rendering health outcomes not merely biological events but socially conditioned processes with cumulative behavioral, symbolic, and physiological consequences.

The correlation between deleterious habitués, particularly tobacco use, alcohol consumption, and areca nut chewing, and the pathogenesis of oral disorders constitutes one of the most prevalent public health concerns in India. These practices, deeply embedded within the cultural, socio-economic, and ritualistic fabric of Indian society, act as potent etiological agents for a range of oral morbidities, most notably oral squamous cell carcinoma (OSCC), oral submucous fibrosis (OSMF), and chronic periodontitis. The epidemiological magnitude of this burden is unparalleled, with India contributing to nearly one-third of the global incidence of oral cancer (194, 197). Tobacco, in both smoked and smokeless forms, remains the predominant behavioral carcinogen. The widespread and extensive use of gutkha, khaini, and zarda (cheaper and more accessible than cigarettes) has rendered smokeless tobacco an endemic affliction across rural and peri-urban India (198). These products are replete with tobacco-specific nitrosamines (TSNAs), reactive oxygen species, and heavy metals that induce epithelial dysplasia, immune dysregulation, and mutagenesis (199, 200). Notably, chronic exposure results not merely in localized mucosal changes but systemic immune-inflammatory shifts that potentiate malignant transformation.

Alcohol, though less culturally ubiquitous than tobacco, acts synergistically with it to exacerbate oncogenic risk. Ethanol serves as a mucosal penetrant, facilitating deeper absorption of carcinogens, while concurrently inducing salivary gland hypo-function, oxidative stress, and microbial dysbiosis; factors that cumulatively destabilize oral homeostasis (201, 202). Despite growing evidence of harm, alcohol consumption is rapidly increasing across socio-economic strata, particularly among young urban males (203). The areca nut, either by itself or in combination with betel quid, is arguably the most culturally sanctioned of these habitués. Its primary alkaloid, arecoline, has been discerned to enhance the traits of OSMF: collagen deposition, fibroblast proliferation, and epithelial atrophy (204, 205). India accounts for an estimated 80% of global OSMF cases, with prevalence particularly high among adolescents and women from low-income communities (206–209). Alarmingly, longitudinal studies report malignant transformation rates in OSMF ranging from 7% to 13%, underscoring the pre-neoplastic potential of habitual areca nut use (210).

Compounding the physiological harms are sociocultural and structural barriers: poor health literacy, stigma, economic dependence on tobacco industries, and a dearth of accessible preventive oral healthcare services. Early signs such as leukoplakia, erythroplakia, and persistent ulcerations are often disregarded or misinterpreted, resulting in late-stage presentation and dismal survival outcomes (211). The National Health Policy (2017) and the MPOWER tobacco control strategy represent incremental progress, yet implementation gaps persist.

Thus, in the Indian milieu, habitués are not mere individual risk behaviors; they are systemic vectors of disease deeply intertwined with caste, class, gender, and economic precarity. Reducing their oral health impact necessitates an integrative strategy combining molecular research, culturally competent education, legislative enforcement, and sustained community-based interventions.

#### 2.3.5 Social determinants of oral health and vitamin D deficiency in India

Oral health in India reflects broader social inequities and lifestyle transitions. The rise of urbanization and Westernized diets has led to increased consumption of sugar-rich and processed foods, contributing to higher prevalence rates of dental caries and periodontal diseases (188). Lifestyle factors, including tobacco use, are intricately linked to oral health disparities. India's socioeconomic gradient influences patterns of tobacco consumption, with marginalized communities disproportionately engaging in smokeless tobacco use due to affordability and cultural acceptability (212). Oral health outcomes are further mediated by access to healthcare services. Government healthcare initiatives often prioritize communicable diseases, leaving oral health inadequately addressed. Financial barriers and cultural stigma around seeking dental care exacerbate these challenges. Moreover, women's oral health outcomes are particularly affected by patriarchal norms that prioritize family welfare over individual health, delaying or neglecting dental treatment (195).

In India, socioeconomic status (SES) is a key determinant of oral health outcomes, with lower SES groups bearing a disproportionate burden of oral diseases due to limited access to care, inadequate health literacy, and higher prevalence of deleterious habits. The study by Chandra Shekhar and Reddy (2011) in Mysore demonstrated significantly higher rates of untreated dental caries, tooth loss, periodontal disease, and oral precancerous lesions among individuals from lower SES backgrounds (213). In contrast, higher SES groups exhibited better oral hygiene practices, increased utilization of dental services, and a greater number of filled teeth, highlighting stark disparities. Complementing these findings, a study from Gwalior district (214) reported that financial constraints are a major barrier to seeking dental care, largely due to the high cost of procedures. Similarly, Deolia et al. found that 12.4% of individuals preferred government-sponsored treatments for their affordability, emphasizing the need for accessible, cost-effective oral healthcare services (215).

Nevertheless, a study of nomads in southern India reveals that oral healthcare is often navigated through the lens of *Stoicism*, a philosophical disposition deeply embedded in their cultural psyche (216, 217). Stoicism valorizes the endurance of pain and discourages its expression, thereby framing help-seeking as a sign of weakness. Simultaneously, fatalism—another prevalent belief—constructs illness as an inevitable fate, rendering medical intervention seemingly futile, especially in the context of life-threatening conditions such as oral cancer (218). These ideologies are compounded by a strong ethic of self-reliance, wherein individuals opt for traditional home remedies over formal dental care. Despite a notable shift toward the use of toothbrushes, ineffective techniques persist, contributing to widespread gingivitis and periodontitis (217). Such outcomes may also be obscured by social desirability and information biases. Ultimately, entrenched misbeliefs and cultural narratives surrounding health and illness act as substantial barriers to accessing care, highlighting the critical need for culturally responsive oral health interventions within nomadic populations. Devi et al. reported that the majority of the Gypsy Narikuravar population in Puducherry demonstrate poor awareness of oral health and hygiene, lacking knowledge of basic practices such as appropriate tooth brushing techniques, the use of toothpaste, and other essential oral hygiene aids (219). This significant gap in oral health literacy highlights a broader systemic neglect of marginalized communities in public health outreach. The data from this study underscore the pressing need for culturally sensitive, community-based oral health education programs tailored to the specific needs of the Narikuravar. In addition, there is a critical demand for the integration of comprehensive and accessible oral healthcare services aimed at preventing and managing periodontal diseases within such underserved populations, thereby promoting long-term oral health and overall well-being.

Concurrently, vitamin D deficiency in India represents a pressing public health issue, with an estimated prevalence exceeding 80% in certain populations (220). Social determinants, including socioeconomic status, education, occupation, and geographical location, play pivotal roles in shaping vitamin D levels. Urbanization, characterized by sedentary lifestyles and indoor-oriented occupations, restricts natural sunlight exposure, exacerbating deficiency risks (71). Moreover, the widespread use of skin-lightening products, driven by societal beauty norms, further limits the cutaneous synthesis of vitamin D. Education levels significantly influence awareness and adoption of preventive measures against vitamin D deficiency. Conversely, populations in rural and underprivileged settings often lack such awareness, compounding their vulnerability. Geographical factors, including latitude and climate, also intersect with sociocultural determinants. Regions with prolonged monsoons or dense urban landscapes experience reduced UV-B radiation, further impacting vitamin D synthesis.

Lifestyle factors form a critical nexus between vitamin D deficiency and oral health. Insufficient sun exposure, dietary inadequacies, and sedentary behaviors contribute to poor bone health and increased susceptibility to periodontal diseases. Vitamin D plays a vital role in calcium metabolism, influencing dental and skeletal health. Deficiency has been implicated in conditions such as periodontitis, underscoring the interdependence between systemic and oral health (221). Cultural dietary practices further exacerbate these interconnections. The predominance of carbohydrate-rich diets in lower-income households, coupled with limited access to calcium and vitamin D sources, contributes to both oral health deterioration and systemic deficiencies. Furthermore, the coexistence of malnutrition and obesity in India-a phenomenon termed the “dual burden of malnutrition”-complicates efforts to address these health issues comprehensively (222).

#### 2.3.6 The invisible deficiencies of fairness, fortification, and the forgotten: an intersectional lens on vitamin D deficiency in caste-stratified India

In India, vitamin D deficiency is shaped not only by biological and environmental factors but also by deep-rooted cultural practices, structural inequalities, and aesthetic ideals. Cultural behaviors around sun exposure are heavily influenced by traditional clothing norms, gendered expectations, and sociocultural beauty standards that favor lighter skin. These factors intersect to limit exposure to ultraviolet B (UVB) radiation, which is crucial for endogenous vitamin D synthesis, primarily among women and higher caste groups (223). The aesthetic valorization of fair skin in India is a legacy of colonialism, caste based stratification, and media reinforcement, resulting in the widespread use of skin-lightening products and sun-avoidant behavior (224). Fairness is frequently equated with virtue, beauty, and social capital, particularly among women, resulting in deliberate avoidance of sunlight through umbrellas, scarves, and long-sleeved clothing (225, 226). Moreover, this concept of aesthetics extends to oral health as well, influencing behaviors and outcomes, particularly among school-aged children. A cross-sectional, multilocal study in Guntur district reported a higher prevalence of dental caries in males compared to females, potentially due to greater aesthetic awareness among girls, prompting them to seek timely treatment such as tooth-colored restorations (227). Enhanced concern for appearance may also contribute to more diligent oral hygiene practices among females, including regular brushing and flossing. The same study found that children who brushed twice daily had a 43.1% caries-free rate, underscoring the importance of consistent oral hygiene routines. However, lower caries-free rates were attributed to inadequate brushing techniques and suboptimal hygiene habits. Complementing these findings, research in Kamrup district, Assam, revealed significantly poorer oral health among rural children and a decline in hygiene with age, with males again exhibiting worse outcomes than females (228). These aesthetic preferences are not merely individual choices but socially sanctioned practices shaped by caste, gender, and class hierarchies. Structural barriers exacerbate these behavioral norms. Despite high national prevalence of vitamin D deficiency, food fortification policies remain inconsistently implemented. The Food Safety and Standards Authority of India (FSSAI) launched the “+F” initiative to promote fortification, yet fortified foods are often inaccessible to low-income and marginalized communities due to cost, distribution, and dietary mismatch (229, 230). Moreover, lactose intolerance and vegetarian diets common among many Indian populations limit intake of natural dietary sources like dairy and fish (229).

Historically, Dalit and tribal groups engaged in agrarian and manual outdoor labor, which might have provided natural protection against deficiency. However, rapid urbanization and extensive migration have led to a shift toward indoor and informal labor markets, reducing sunlight exposure even among these groups. A national study by Harinarayan et al. reported that over 70% of Indian adults exhibited suboptimal serum 25(OH)D levels, with disproportionately higher deficiency among women, urban poor, and lower caste groups (223). Additionally, limited awareness and low health literacy regarding vitamin D's role in health significantly obstruct preventive efforts, particularly among socially marginalized groups. Public health campaigns often fail to effectively engage non-dominant language speakers, rural populations, and lower-caste communities due to linguistic, cultural, and infrastructural barriers (231). This communication gap is further exacerbated by historical patterns of systemic exclusion from healthcare services, unfortunately, which have left these groups under-informed and underserved. Preventive interventions such as vitamin D screening, supplementation, and counseling are predominantly concentrated in urban centers and among socioeconomically privileged populations. As a result, lower caste and rural individuals are less likely to receive timely diagnoses or benefit from state-supported nutritional programs. The convergence of structural neglect and social marginalization perpetuates a cycle of deficiency and reinforces broader health disparities across caste and class lines.

In totality, vitamin D deficiency in India reflects more than a public health concern which reveals a strong interconnection of caste-based discrimination, aesthetic hegemony, and structural neglect. Tackling this requires intersectional strategies that address not only biochemical needs but also the sociocultural ideologies underpinning sun exposure behaviors.

### 2.4 Ethnomedicinal approaches to oral health and vitamin D deficiency in India

Ethnomedicine, as a discourse, explores traditional medical systems within the cultural and ecological tapestry of India. Here, ethnomedicine encompasses Ayurveda, Siddha, Unani, Naturopathy and various indigenous practices rooted in regional knowledge systems. These traditions offer health solutions, which accord with the cultural construct particularly of the rural and underserved populations. The ethnomedical approach integrating natural remedies, dietary guidelines, and lifestyle modifications, induce a symbiotic relationship between individuals and their environment (232). The Indian discernment of ethnomedicine is highly intertangled with the biodiversity of the subcontinent. Medicinal plants such as Ashwagandha (*Withania somnifera*), Tulsi (*Ocimum sanctum*), and Amlaki (*Emblica officinalis*) are revered for their therapeutic properties (233). These medicinal plants are rich in bioactive compounds that promote immunity, metabolic health, and tissue regeneration, improving certain conditions such as vitamin D deficiency and oral diseases (234). In traditional sacred texts like Ayurveda, the notion of *dinacharya* (daily routine) and *ritucharya* (seasonal regimen) promotes sustainable health practices. These routines emphasize sun exposure, dietary balance, oral hygiene, and physical activities, factors intricately linked to vitamin D levels and oral health. For instance, practices such as oil pulling (*kavala* or *gandusha*) using sesame or coconut oil are recommended for maintaining oral hygiene and preventing systemic diseases (235). In the context of modern health challenges such as oral health disparities and vitamin D deficiency, the principles of traditional healing knowledge system, combined with contemporary medicine offer transformative potential for combating these pervasive issues in India.

Different traditional health care system and ethnomedicine offer pathways to mitigate vitamin D deficiency by emphasizing sunlight exposure, dietary interventions, and lifestyle modifications.

• Herbal Supplementations: Sacred texts’ pharmacopeia includes herbs that improve gut health and metabolic functions, thereby optimizing nutrient absorption. For example, Guduchi (*Tinospora cordifolia*) and Shatavari (*Asparagus racemosus*) are known to enhance overall vitality and support musculoskeletal health, addressing complications associated with vitamin D deficiency (233, 236).
• Dietary Propositions: It also advocate the consumption of seasonal and regionally available foods. Incorporating vitamin D-rich ingredients such as ghee, fortified milk, and fish into daily diets aligns with traditional dietary principles. Additionally, Amlaki, known for its high vitamin C content and provides antioxidant stimulant, enhances calcium absorption, indirectly supporting bone health (237).
• Sunlight and Surya Namaskar: The ancient sacred texts extol the importance of sunlight as a vital source of *Prana* (life energy). Practices such as *Surya Namaskara* (sun salutation) not only enhance physical fitness but also facilitate natural sunlight exposure, aiding endogenous vitamin D synthesis (238). These practices align with culturally familiar routines, encouraging sustainable adoption.

Oral health has long been a focal point in Indian traditional healthcare knowledge and practices. The use of natural substances for dental care not only promotes hygiene but also aligns with sustainable and culturally relevant practices.

• *Dantadhavana*: Traditional healing practices recommend brushing teeth with herbal twigs, such as those from neem (*Azadirachta indica*) or babool (*Acacia nilotica*). These twigs are chewed at one end to form a brush-like texture, releasing natural antimicrobial agents that inhibit biofilm buildup and gingival diseases (239).
• The Multifaceted Aid: Neem, often referred to as the “village pharmacy,” possesses potent antibacterial, antifungal, and anti-inflammatory properties. Regular use of neem twigs for brushing not only ensures oral hygiene but also prevents systemic infections by reducing bacterial load (240). Neem-based toothpastes and powders have gained popularity for their efficacy in managing gingivitis and periodontitis.
• Oil Pulling: Oil pulling (*Kavala* or *Gandusha*) involves swishing sesame or coconut oil in the mouth for several minutes to eliminate toxins and improve oral hygiene. Studies have shown that oil pulling reduces oral microbial count, thereby preventing caries and halitosis (235). This practice also aligns with contemporary comprehensions of the oral-systemic health connection.
• Herbal Toothpaste: Ancient approaches such as herbal toothpaste and DantKanti, which include a combination of herbs like Neem (*Azadirachta indica*), clove (*Syzygium aromaticum*), liquorice (*Glycyrrhiza glabra*), cinnamon (*Cinnamomum verum*), turmeric (*Curcuma longa*) Anantmul (*Hemidesmus indicus*), Vajradanti (*Barleria prionitis*), and mint, are used for strengthening teeth and gums. These formulations provide anti-diabetic, antimicrobial and anti-inflammatory properties, supporting all-inclusive oral health and hygiene (241).

The traditional oral health care practices in India provide a compelling integrative framework for preventive oral health strategy. Vitamin D, particularly in its salivary form, is increasingly recognized as a non-invasive biomarker indicative of both systemic and oral immunity, maintain calcium homeostasis, facilitating antimicrobial defence, and modulating periodontal inflammation. The ethnomedicinal interventions, including medicinal plants such as neem (*Azadirachta indica*) or babool (*Acacia nilotica*), Tulsi (*Ocimum sanctum*), Amla (*Emblica officinalis*) have demonstrated significant antioxidant, antimicrobial and anti-inflammatory properties, which are well documented to reduce oral pathogens, suppress gingival inflammation and enhance oral health in general and create a more favorable oral microenvironment for optimal efficacy in local immunomodulatory function of vitamin D (239, 240). Various traditional interventions specifically oil pulling with coconut or sesame oil can mitigate local microbial invasion and growing biofilm. It can attenuate inflammatory burden by reducing oxidative stress and increase the bioavailability of vitamin D within periodontal tissues (235). Moreover, the consistent use of phytochemicals such as polyphenols, flavonoids, and eugenol present in neem and clove may regulate cytokine profiles, modulate microbial flora and sustain epithelial integrity, thus optimizing the tissue environment in which the activity of vitamin D dependent pathways involved in immunoregulation and bone homeostasis can operate to its full potential (240). Although there is paucity of current literature that can glean the conclusive and direct evidence of these traditional modalities elevating the level of salivary vitamin D, these are emerged as auxiliary avenue to facilitate the optimal function of vitamin D and support periodontal health through synergistic mechanisms.

While traditional healthcare systems such as Ayurveda, Unani, and ethnomedicine offer culturally resonant modalities of care, their efficacy in addressing the complicated associations between oral health and vitamin D remains circumscribed. These systems often emphasize humoral balance, dietary customs, and herbal therapeutics, yet lack standardized protocols or empirical grounding in micronutrient biology, specifically in relation to vitamin D synthesis and its oral-systemic implications. Moreover, their epistemological frameworks rarely incorporate contemporary comprehensions of periodontal pathology, bone metabolism, or immune modulation, thereby limiting their relevance for contemporary preventive care. Despite their socio-cultural accessibility and deep-rooted acceptance, traditional approaches of folk-medicine or ethnomedicine may inadvertently perpetuate fragmented care when not aligned with biomedical diagnostics or public health infrastructures. Future integration must prioritize interdisciplinary models that synergize traditional knowledge with biomedical science through community-based initiatives, clinical validation of traditional formulations, and incorporation of oral health and micronutrient awareness into AYUSH education. Implementation should be rooted in policy frameworks that enable pluralistic health delivery. This may include embedding AYUSH practitioners within primary healthcare teams, developing unified guidelines for micronutrient supplementation, and institutionalizing cross-referral systems to ensure continuity of health care. Such integrative approaches can enhance cultural competence, bridge care disparities, and promote holistic well-being.

### 2.5 Socio-cultural context and structural solutions: potential public health policy pathways in India

A socio-cultural perspective can shed light on the machinery of structural inequalities in effectuating health disparities. In India's socio-economic and socio-political landscape, intersectionality of caste, class, and gender emerges to be instrumental for disproportionate health outcomes and access to care. Vitamin D deficiency and oral health disparities, rooted in lifestyle factors and social determinants, underscore the need for comprehensive public health strategies. Policymakers can alleviate these expansive issues and ensure holistic well-being by focusing on the socio-cultural determinants of health practices and facilitating equitable access to healthcare. Public health interventions must resolve these perpetual barriers by incorporating approaches that align with the socio-cultural fabric of people.

Despite India's strides in improving its dentist-to-population ratio, which improved from a staggering 1: 300,000 in the 1960s to approximately 1: 10,000 by 2010 (242), the pursuit of equitable oral healthcare remains elusive. The rural-urban dichotomy remains stark, with rural regions suffering an appalling 1: 250,000 ratios, underscoring the systemic neglect of underprivileged populations (243). This inequity is further intensified by the clustering of dental professionals within urban private practices, which are financially out of reach for vast segments of the population. The structural inadequacies of public healthcare infrastructure, particularly the absence of dental posts in rural Primary Health Centres (PHCs), restrict not only curative but also preventive services. Empirical evidence from Tamil Nadu illustrates the extent of these challenges. In Chengalpattu, 91.5% of rural inhabitants identified access barriers rooted in cost, infrastructural insufficiency, and procedural anxiety (244). These findings reveal how poor health-seeking behavior, compounded by limited oral health literacy, perpetuates untreated morbidity. To rectify this, scalable, community-rooted strategies such as mobile dental units, school-based screenings, and routine dental camps are critical. Such models must align with the expressed priorities of rural communities and emphasize primary prevention (215).

Although the Indian Dental Association (IDA) proposed a National Oral Health Policy as early as 1986, which was subsequently endorsed in 1995, it has languished without substantial implementation (245). Consequently, India's oral health landscape remains fragmented, marked by infrastructural insufficiencies and inequitable distribution of care. The proliferation of dental colleges, while positioning India as a global leader in dental education, has not translated into rural service delivery improvements (246, 247). Recommendations by the Planning Commission to reform curricula and embed community integration in dental education have largely gone unheeded, leaving departments of community dentistry siloed within urban hospital settings (248–250). A deeper workforce crisis looms, driven by geographic maldistribution, surplus production of underemployed dental graduates, and a stark paucity of specialists and auxiliaries in rural and non-urban settings (251). These issues are exacerbated by India's minimal 2% health budget allocation, of which an even smaller portion addresses oral health (252). Moreover, the average percentage of total health expenditure in relation to GDP has remained relatively low, specifically at (3.64 ± 0.43) %, reflecting a systemic underinvestment in healthcare infrastructure and human resources (253). Key demographic shifts, especially ageing populations, warrant urgent investment and implementation in geriatric dentistry, which is currently a glaring blind spot in policy, education, and infrastructure (254).

Oral diseases, though largely preventable, contribute to significant economic loss, particularly among informal and daily wage laborers (255). Unregulated quackery and unsafe dental practices remain widespread due to lax enforcement and public desperation (256). Innovative outreach models like mobile clinics and portable dental units, though promising, suffer from policy neglect and lack of institutional backing (257). Public–private partnerships (PPP), while conceptually robust, remain poorly regulated and disproportionately benefit urban centres (258, 259). Moreover, voluntary accreditation fails to enforce minimum standards across dental education institutions (260). Policy reorientation must draw from global exemplars such as Brazil's Unified Health System or Germany's Social Health Insurance, where oral health care is integral to the primary health care and overall health insurance frameworks (261). Embedding dental personnel in every PHC and CHC, developing national oral health surveillance systems, and promoting water fluoridation and culturally contextualized education are imperative (262–264).

Simultaneously, vitamin D deficiency, which remains massively prevalent across diverse population groups in India, presents a significant public health challenge due to its established associations with both oral and systemic health outcomes. Hypovitaminosis D has been linked to periodontal disease progression, impaired bone mineralization, delayed tooth eruption, and compromised immune function, underscoring its importance within the oral health framework. Addressing this widespread deficiency requires the implementation of a comprehensive Vitamin D Supplementation Program of India (VDSPI). This initiative should adopt a multi-faceted approach encompassing widespread public education, culturally sensitive campaigns promoting safe sun exposure, and targeted, evidence-based supplementation strategies (265). Incorporating vitamin D screening into routine primary healthcare services, particularly for high-risk groups such as children, pregnant women, and the elderly, would further strengthen early detection and preventive care. Pilot programs in metropolitan cities, supported through collaborative efforts involving government agencies, civil society, and healthcare providers, can offer scalable models for national adoption. Integrating these programs within broader preventive and community health frameworks will help reduce the burden of deficiency-related diseases and enhance equity and resilience in oral healthcare delivery.

Furthermore, health programs must integrate traditional healthcare system and medicine with contemporary medicine to provide an inclusive, equitable and holistic preventive and curative services, ensuring affordability and accessibility for marginalized groups. Community-based interventions capitalizing on traditional knowledge can yield trust and promote sustained behavioral change. Priority should be given to public awareness campaigns for vitamin D deficiency, emphasizing the importance of sunlight exposure and dietary diversification. Fortification of staple foods, such as milk and edible oils, offers a scalable solution to tackle deficiencies across diverse populations (100). Here, traditional healthcare system and ethnomedicine can suffice to minimize health inequalities by leveraging local resources and culturally compatible practices, since these systems have got enormous sociocultural relevance for their accessibility and acceptance among Indian populations. For instance, incorporating traditional diets and oral hygiene practices within community health programs can conciliate between awareness and access. Public health initiative, integrating ethnomedicine with modern healthcare systems, can enhance health outcomes. Policies promoting cultivation and use of medicinal plants along with public education and awareness initiatives, can grapple with vitamin D deficiency and oral health disparities in a sustainable manner. Symbiosis of traditional healers and biomedical practitioners can create inclusive and culturally competent holistic care model (266), which also provide insightful discernment for oral health inequalities as well as vitamin D deficiency in India. These systems produce sustainable solutions to contemporary health challenges, by focusing on the inclusive, equitable practices appertain to cultural framework of populations. The amalgamation of traditional dietary practices, herbal remedies, and oral hygiene techniques with modern healthcare can provide systemic liaison, promote equity in health outcomes and reaffirm the ingrained association between culture and well-being.

**Figure 4:**
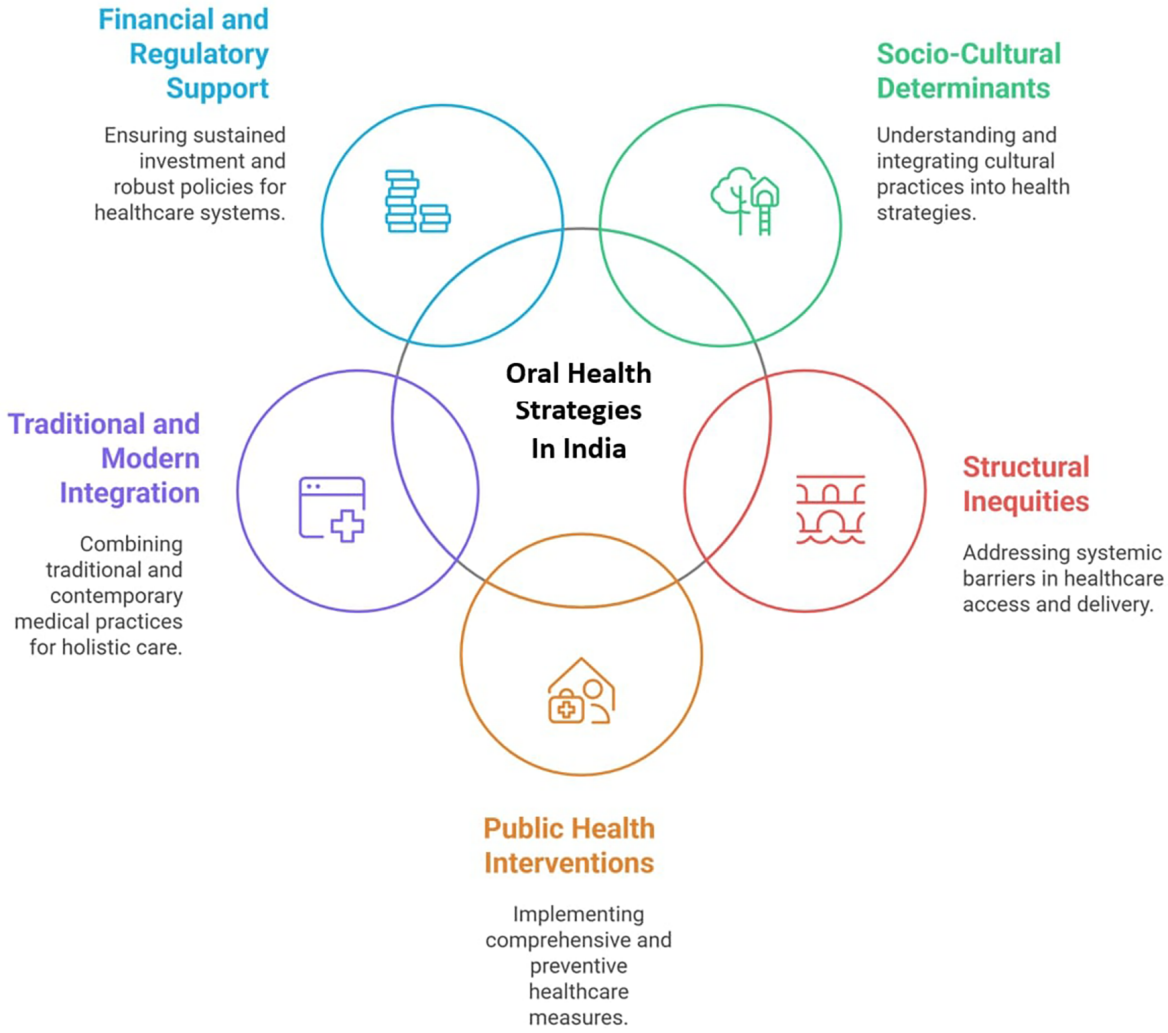
Potential policy interventions to provide comprehensive oral health strategies in India.

Ultimately, the realization of India's broader health and developmental aspirations hinges on the adoption of holistic, comprehensive, prevention-focused, and equity-driven health strategies (Figure 4). These must be supported by sustained financial investment, rigorous regulatory frameworks, and cohesive multi-sectoral collaboration to ensure long-term impact and systemic transformation.

## 3 Conclusion

Oral health and vitamin D deficiency, though often sidelined in public health discourse, are deeply interconnected challenges that demand urgent, culturally grounded solutions in India. These issues are not merely biomedical but are intricately shaped by broader socio-cultural determinants, including lifestyle practices, health behaviors, and structural inequalities. The concept of habitus, shaped by social norms, cultural beliefs, and environmental exposure, offers a valuable framework to understand and influence these determinants, particularly in the Indian context where traditional knowledge systems continue to shape health perceptions and practices. Despite India's geographic advantage for sunlight exposure, the rising prevalence of vitamin D deficiency reflects modern behavioral shifts such as reduced outdoor activity, changing dietary patterns, and urbanized living, which must be addressed through preventive strategies. Concurrently, the burden of oral diseases, especially in under-resourced regions, is intensified by inadequate infrastructure, lack of workforce distribution, and minimal integration of oral health into primary healthcare services. To bridge these gaps, public health interventions should leverage culturally familiar and community-based approaches. Integrating traditional dietary practices, natural oral hygiene methods, and moderate sun exposure, validated through contemporary research, into national health strategies can enhance both acceptance and effectiveness. For instance, the development of a nationwide Vitamin D Supplementation Program of India (VDSPI), coupled with school- and community-based oral health education, could address dual deficiencies efficiently. Policy frameworks must move beyond abstract rhetoric and adopt concrete steps such as embedding dental professionals in every PHC and CHC, mandating routine vitamin D screening for high-risk groups, fortifying staple foods with essential nutrients, and promoting health literacy through vernacular outreach. Importantly, traditional health systems such as Ayurveda and local ethnomedicine should be systematically evaluated and incorporated where evidence aligns, enabling inclusive and context-sensitive care. Collaboration between biomedical practitioners and traditional healers, supported by public and private partnerships, can foster culturally competent models of care delivery. Programs piloted in urban areas should be scaled to rural settings with the help of mobile clinics and digital health platforms to ensure that no population is left behind.

Ultimately, achieving equitable oral and systemic health in India requires a paradigm shift that transcends disciplinary silos and embraces the synergy of modern science, traditional wisdom, and socio-cultural realities. Through sustained funding, rigorous regulation, and inclusive policy design, India can not only address current health disparities but also establish a resilient and future-ready healthcare system that honors its pluralistic heritage while meeting contemporary needs.
